# Risk factors analysis for extubation failure following mandibular distraction osteogenesis in infants with Pierre Robin sequence: a retrospective cohort study

**DOI:** 10.3389/fped.2026.1806828

**Published:** 2026-05-25

**Authors:** Wenmin Yang, Yingqiu Cui, Feiyan Chen, Chunmin Zhang, Jinda Huang, Hongyan Peng

**Affiliations:** 1 Pediatric Intensive Care Unit, Guangzhou Women and Children’s Medical Center, Guangzhou Medical University, Guangzhou, China; 2 Department of Stomatology, Guangzhou Women and Children’s Medical Center, Guangzhou Medical University, Guangzhou, China

**Keywords:** airway extubation, infant, mandibular distraction osteogenesis, Pierre Robin sequence, pneumonia, risk factors

## Abstract

**Background:**

Mandibular distraction osteogenesis is an important therapeutic approach for infants with Pierre Robin sequence (PRS). There is a lack of data describing risk factors for postoperative extubation failure (EF) in these infants. We aimed to identify risk factors for EF in infants with PRS and to explore the association between EF and clinical outcomes.

**Methods:**

A retrospective cohort study was carried out on infants with PRS who received mandibular distraction osteogenesis at our facility from January 2020 to December 2023. Extubation failure was defined as the need for non-invasive ventilation or reintubation within 24 h of extubation. Multivariate logistic regression analysis was performed to explore the risk factors for EF. Univariable statistical analysis was performed to determine associations between EF and clinical outcomes.

**Results:**

Of 135 included infants, 27 (20.0%) experienced EF. In the logistic regression analysis, postoperative pneumonia was independently associated with EF (*p* = 0.029). Compared to those without pneumonia, patients with postoperative pneumonia had a odds ratio (OR) of 5.25 for EF (95% CI, 1.18–23.28; *p* < 0.05). Mandibular distraction length, laryngeal edema, and inflammatory markers (WBC, CRP, temperature) were not associated with EF. Infants who failed extubation had longer durations of mechanical ventilation [5 days (IQR 4–7) vs. 4 days (IQR 4–5), *p* < 0.001], longer PICU lengths of stay [9 days (IQR 8–13) vs. 6 days (IQR 5–6), *p* < 0.001], and longer hospital lengths of stay [18 days (IQR 14–22) vs. 15 days (IQR 13–17), *p* = 0.001] than successfully extubated infants.

**Conclusions:**

Postoperative pneumonia is associated with an increased risk of failing extubation in infants with PRS. Prospective studies are needed to evaluate whether screening for postoperative pneumonia and evaluating airway resistance before extubation can reduce the risk of EF.

## 1 Introduction

Pierre Robin sequence (PRS) is a triad of micrognathia, glossoptosis, and airway obstruction (1, 2). These clinical features arise from mandibular hypoplasia, which leads to posterior displacement of the tongue (glossoptosis) and consequent pharyngeal obstruction with respiratory compromise (3). In recent years, mandibular distraction osteogenesis (MDO) has been adopted by many centers as the primary surgical intervention after conservative methods of relieving airway obstruction have failed (4–6). Through MDO, the tongue is repositioned forward, which helps alleviate airway obstruction. After MDO, all infants still need endotracheal intubation and mechanical ventilation (MV) in the pediatric intensive care unit (PICU) for a period of time. In our clinical practice, we have observed that infants with PRS have a higher incidence of postoperative extubation failure (EF). Previous studies have shown that EF prolongs MV duration and increases mortality in ICU patients (7, 8). Therefore, identifying risk factors for EF and implementing early interventions are critical for reducing EF.

At present, limited data exist on the risk factors of EF following MDO in infants with PRS. While we have frequently observed postoperative pneumonia in these infants in our PICU, it remains unclear whether postoperative pneumonia is a risk factor for EF. Only one study suggested that pneumonia at the time of weaning was associated with post-weaning hypoxemia in PRS infants (9). Our study aims to retrospectively analyze the clinical data of infants with PRS who underwent MDO at the Guangzhou Women and Children's Medical Center, and to explore the risk factors and outcomes of EF. Beyond the standard measures of EF, this study specifically investigated whether postoperative pneumonia is an important risk factor for EF following MDO.

## 2 Materials and methods

### 2.1 Design, setting, and patients

This single-center retrospective cohort study involved all infants diagnosed with PRS who received MDO due to upper airway obstruction (UAO) at the Guangzhou Women and Children's Medical Center in Guangzhou, China, between January 2020 and December 2023. The Ethical Committee of the same medical center granted a waiver for the study's approval. Informed consent was not needed for patients because of the retrospective design of the study, as determined by the Ethical Committee. The diagnosis of PRS was based on discharge diagnosis recorded in the electronic medical records (EMRs). MDO was primarily reserved for infants that experienced significant airway obstruction refractory to non-invasive treatments, such as prone positioning and nasopharyngeal airway placement. Severe airway obstruction was defined as having at least three clusters of desaturations, with a minimum of three dips below 80% within a 24-hour period. Additionally, CT scans and fiber-optic bronchoscopy were utilized to examine airway obstruction severity. The inclusion criteria included: (1) patients with a diagnosis of PRS, (2) treatment with MDO, (3) age between 1 and 12 months, and (4) all subjects underwent standard endotracheal intubation and MV as part of the preoperative process in the operating room on the day of surgery, followed by transfer to the PICU with tracheal tube. The exclusion criteria were as follows: (1) age exceeding 1 year, (2) presence of chronic lung disease, (3) history of previous mandibular distraction osteogenesis, and (4) incomplete data.

All operations were performed by the same group of surgeons. Bilateral mandibular osteotomies were carried out, with distractors bilaterally affixed using screw fixation. The direction of the traction was carefully aligned along the condylar line, extending toward the mental point. Initially, the mandibular angle was subjected to distraction of 1.4–2.1 mm over a period of 3 days, with adjustments made twice daily—0.7–1.05 mm in the morning and evening. Following this initial phase, a consistent daily distraction of 1.40 mm was applied, again divided into two sessions of 0.70 mm each in the morning and evening. This methodical approach continued until the patient exhibited a prognathism. Ultimately, a distraction device (Zhongbang, Xi'an, Shanxi, China) measuring between 10 and 20 mm was strategically positioned at the mandibular angle.

### 2.2 Weaning and extubation protocol

Pressure-controlled ventilation served as the primary ventilatory modality. Weaning and extubation adhered to institutional PICU protocols. Typically, the following criteria must be met before weaning: (a) a postoperative distraction of greater than 5 mm lasting at least 3 days; (b) sufficient gas exchange and ventilation, as evidenced by SpO_2_ ≥94% while the patient is on the fraction of inspired oxygen (FiO_2_) of ≤0.40 and PEEP of ≤5 cmH_2_O; (c) adequate respiratory drive; (d) present cough reflexes; (e) appropriate level of consciousness; and (f) hemodynamic stability without vasoactive support. Furthermore, the intensive care physician must concur that the patient is stable and prepared for extubation.

Participants meeting the above weaning criteria underwent a 15-minute spontaneous breathing trial (SBT). During this period, they were disconnected from the ventilator and breathed spontaneously through a flow-inflating anesthesia bag under physician supervision (10). Those who completed the SBT were extubated. The criteria for SBT failure included the following: (a) increased respiratory rate (for infants under 6 months, respiratory rate >60/min; for those aged 6 months to 1 years, respiratory rate >45/min; (b) indications of increased respiratory effort (such as utilization of accessory respiratory muscles); (c) heart rate >160 bpm; and (d) SpO_2_ <90%.

For patients requiring prolonged mechanical ventilation (≥7 days), intravenous dexamethasone was administered peri-extubation in divided low-dose regimens, specifically 12 h before and 12 h after extubation.

### 2.3 Post-extubation therapies and EF definitions

Nebulized epinephrine was routinely administered immediately after extubation for all patients to reduce laryngeal edema. In cases where patients exhibited respiratory dysfunction following extubation, prompt interventions were implemented, including repeated nebulization of β-agonist therapy and concurrent airway clearance/cough augmentation strategies. If clinical conditions continued to deteriorate, NIV or reintubation was instituted in the presence of any of the following: changes in pH or PaCO_2_; SpO_2_ <90% despite an FiO_2_ greater than 0.5; or increased signs of respiratory distress (e.g., utilization of accessory respiratory muscles, tachypnea, and the thoracoabdominal paradox). The main endpoint was extubation failure, which was defined as the requirement for reintubation or non-invasive ventilation within 24 h after extubation (11). Patients were categorized into successful extubation and failed extubation groups accordingly. Only the first extubation attempt was included for patients with multiple failures.

### 2.4 Data collection and statistical analysis

A single investigator reviewed the EMRs to extract demographic information, clinical data, and extubation outcomes. The demographic factors included age, sex, body weight, and discharge diagnosis. The clinical information included both preoperative and postoperative data. Preoperative factors consisted of the highest axillary temperature, white blood cell count (WBC), presence of rales or rhonchi, radiographic infiltrate, and preoperative pneumonia. Postoperative factors included pre-extubation ventilatory and physiological parameters, positive oral secretion cultures, postoperative pneumonia, peak body temperature, WBC counts, levels of C-reactive protein (CRP), distraction length at the time of extubation, and occurrences of post-extubation stridor (PES). Pre-extubation ventilatory and physiological parameters were characterized by the last documented values prior to extubation. These parameters comprised PaO_2_, PaCO_2_, FiO_2_, the PaO_2_ to FiO_2_ ratio, peak inspiratory pressure (PIP), PEEP, tidal volume, respiratory rate, axillary temperature, CRP, and WBC counts. Post-extubation laryngeal edema was assessed by post-extubation stridor. Post-extubation stridor is typically described as a high-pitched wheeze during inspiration caused by airflow limitation from laryngeal narrowing (12).

Postoperative pneumonia was identified through two sources: (1) discharge diagnoses recorded in the EMRs, and (2) manual EMRs review. For cases identified via manual EMRs review, pneumonia was defined by fulfillment of the following three criteria (13): (1) Chest radiological examination (plain radiograph) shows any of the following: infiltrate, opacity, consolidation, or cavitation. All radiographs were obtained via a Toshiba KXO-50R DR system and independently reviewed by two radiologists and senior critical care physicians. (2) It includes at least one criterion: (a) Fever >38 °C without alternative etiology; (b) Leukopenia (<4 × 10^9^/L) or leukocytosis (≥12 × 10^9^/L); (3) It requires one or more of the following: positive quantitative culture from lower airway samples; or new onset of purulent sputum, or changes in sputum characteristics, or increased respiratory secretions, or greater suctioning needs; or new onset or worsening respiratory symptoms (cough, tachypnea, dyspnea, rales/rhonchi), or deteriorating oxygenation (e.g., O_2_ desaturations, increased oxygen or ventilator requirements).

The primary outcome parameters comprised ventilator days, PICU lengths of stay (LOS), and total hospital stay. The outcomes were compared between patients with failed extubation and successful extubation.

Risk factors for EF were identified through multivariable logistic regression, preceded by univariable screening of candidate variables. Continuous data were described as mean ± SD or median (IQR) following Shapiro–Wilk normality assessment. Two-group comparisons utilized two-sample *t*-test for normally distributed variables and Mann–Whitney *U* test for non-normal variables. Categorical variables were presented as counts and percentages. Fisher's exact test or the chi-square test was applied to assess univariable associations between categorical variables. Variables with *p* < 0.10 in the univariable analysis were selected as candidate variables for the multivariable model. These candidate variables were initially entered simultaneously into a multivariable logistic regression model. Collinearity among these variables was assessed using variance inflation factors (VIF); a VIF >10 was considered indicative of severe multicollinearity, and such variables were subsequently removed, and the model was refitted with the remaining variables. Associations between risk factors and EF are reported as odds ratios (ORs) with 95% confidence intervals (CI). Univariable analyses were conducted to assess the relationships between EF and clinical outcomes. All the statistical tests were two-tailed, with *p* ≤ 0.05 deemed statistically significant. Analyses were carried out with SPSS Statistics 23.0 (IBM).

## 3 Results

### 3.1 Patient characteristics

**Figure 1:**
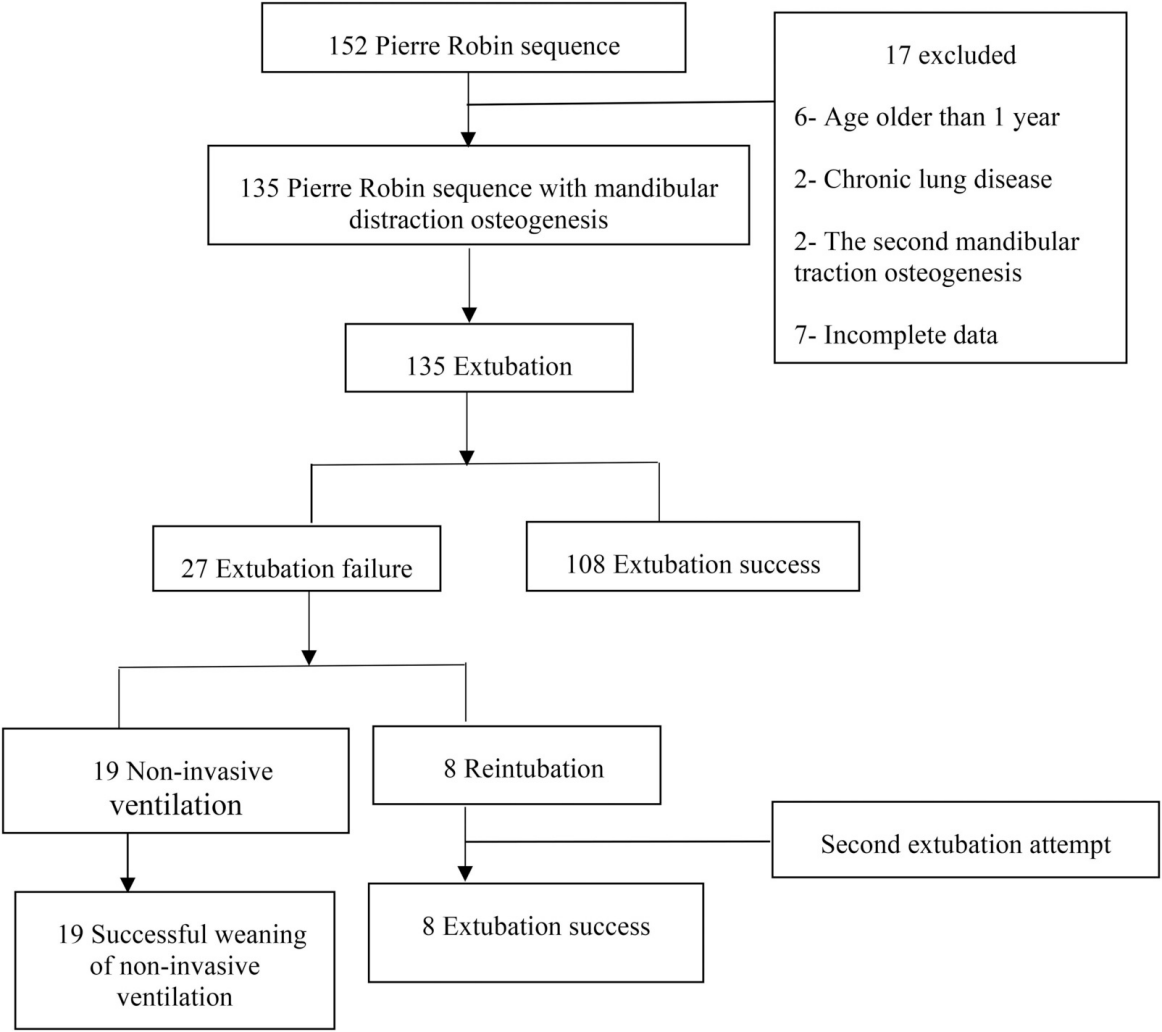
Flow diagram of patient enrollment and extubation outcomes. Flow diagram of patient enrollment and extubation outcomes): In the 7 infants with incomplete data: 3 did not have chest x-ray results, 1 did not undergo CRP testing, 2 had unrecorded post-extubation laryngeal edema, and 1 lacked recorded tidal volume values.

Of 152 PRS infants who underwent MDO, 17 were excluded for the following reasons: age >1 year (*n* = 6), chronic lung disease (*n* = 2), second repeat MDO (*n* = 2), or incomplete data (*n* = 7). Seven patients had incomplete data: missing chest x-ray results (*n* = 3), CRP testing (*n* = 1), documentation of post-extubation laryngeal edema (*n* = 2), or tidal volume recordings (*n* = 1). The final study cohort comprised 135 patients. All participants underwent conventional preoperative endotracheal intubation in the operating room. The median duration from the initial intubation to first extubation attempt was 4 days (IQR, 4–5). EF occurred in 27 patients (20%) after the first attempt. Among the 27 cases of EF, there were 19 cases needing non-invasive ventilation and 8 cases needing reintubation who were successfully extubated in the second extubation attempt. All individuals with failed initial extubation were ultimately successfully weaned to room air. Figure 1 depicts patient enrollment and extubation outcomes.

The median age within the study population was 3 months (IQR 1–5; total range 1–12 months), and 65 patients (48%) were male. Active distraction was carried out for a median of 7 days (range, 5–11) and the median total distraction length was 15 mm (range, 12–20 mm). The median distraction length before extubation was 7 mm (IQR, 7–8.4 mm). The patients had a median duration of mechanical ventilation of 4 days (IQR 4–5), a median ICU stay of 6 days (IQR 5–8) and a median hospital stay of 15 days (IQR 13–18). Among the patients with EF, the interval from extubation to reintubation or non-invasive ventilation ranged from 0.5 to 24 h (median, 3 h; IQR, 2–8). No accidental extubations or mortalities occurred in this series.

Postoperative pneumonia developed in 22 patients, among whom 11 cases were (40.7% of the 27 patients who experienced extubation failure) in the failed extubation group. Radiographic imaging revealed infiltrates in all pneumonia patients, most frequently in the right lower lobe; consolidation was noted in one patient, and neither opacity nor cavitation was observed.

### 3.2 EF-associated variables

Potential risk factors for EF were evaluated through univariable analyses, with the results presented in Tables 1, 2. As illustrated in Table 1, preoperative factors were not significantly related to EF (*p* > 0.05). Table 2 shows that the postoperative predictors of EF comprised positive oral secretion cultures, WBC counts, postoperative pneumonia, maximum temperature >38.0℃, and CRP > 20 mg/L (all *p* < 0.05). The distraction length at extubation was greater among EF patients (8.4 mm [IQR 7–10.5] vs. 7 mm [IQR 7–8.4]; *p* = 0.005). No additional variables achieved statistical significance (Table 2).

**Table 1:** Univariable analysis of EF-associated preoperative factors.

Variables	Extubation success	Extubation failure	*p* value
	(*n* = 108)	(*n* = 27)	
Highest axillary temperature >38.0℃, no. (%)	2 (1.9)	0 (0)	1.000
White blood cell count <4 or ≥12 × 10^9^/L, no. (%)	14 (13)	4 (14.8)	0.758
Rales or rhonchi, no. (%)	2 (1.9)	1 (3.7)	0.491
Infiltrate on chest radiological examination, no. (%)	9 (8.3)	2 (7.4)	1.000
Preoperative pneumonia, no. (%)	5 (4.6)	1 (3.7)	1.000

No. (%), frequency with column percentage value.

**Table 2:** Univariable analysis of EF-associated postoperative factors.

Variables	Extubation success	Extubation failure	*p* value
	(*n* = 108)	(*n* = 27)	
Male sex, no. (%)	50 (46.3)	15 (55.6)	0.389
Age, months (median, IQR)	2.5 (1–5)	3 (1–4)	0.586
Pre-extubation ventilatory and physiologic parameters			
PaCO_2_ (mm Hg) (mean, std)	40.8 (7.7)	41.7 (7.3)	0.584
PaO_2_ (mm Hg) (median, IQR)	120.8 (97.5–154.5)	131.3 (111–152.3)	0.340
FiO_2_ (median, IQR)	0.30 (0.30–0.35)	0.30 (0.30–0.35)	0.622
P/F ratio (median, IQR)	392.5 (317.5–472.2)	427.5 (345–507.5)	0.262
TV/weight (mL/kg) (median, IQR)	10.0 (9.1–10.6)	10.0 (9.0–11.0)	0.643
PEEP, cmH_2_O (median, IQR)	4 (4–5)	5 (4–5)	0.563
PIP cmH_2_O (median, IQR)	17 (16–19)	17 (16–18)	0.839
Respiratory rate (median, IQR)	30 (28–32)	30 (28–30)	0.219
Axillary temperature (median, IQR)	36.9 (36.7–37.4)	36.9 (36.8–37.4)	0.392
CRP (median, IQR)	12.02 (5.99–23.4)	9.42 (4.30–20.9)	0.954
WBC counts (×10^9^/L) (median, IQR)	7.1 (5.4–9.1)	6.8 (5.8–10.0)	0.690
Positive culture of oral secretions, no. (%)	3 (2.8)	5 (18.5)	0.008
Postoperative pneumonia, no. (%)	11 (10.2)	11 (40.7)	<0.001
Highest temperature >38.0℃, no. (%)	51 (47.2)	22 (81.5)	0.001
CRP > 20 mg/L, no. (%)	50 (46.3)	19 (70.4)	0.025
Postoperative WBC count <4 or ≥12 × 10^9^/L, no. (%)	39 (36.1)	15 (55.6)	0.065
Length of distraction at extubation, mm (median, IQR)	7 (7–8.4)	8.4 (7–10.5)	0.005
Post-extubation stridor, no. (%)	73 (67.6)	22 (81.5)	0.157

No. (%), frequency with column percentage value; IQR, interquartile range; FiO_2_, fraction of inspired oxygen; P/F ratio, a ratio of PaO_2_ to FiO_2_; TV, tidal volume; PEEP, positive end-expiratory pressure; PIP, peak inspiratory pressure; CRP, C-reactive protein; WBC, white blood cell.

Five candidate variables (postoperative pneumonia, WBC counts, CRP, positive oral secretion culture, and maximum temperature >38.0℃) were initially entered into the multivariable model. CRP (VIF = 19.1) and maximum temperature >38.0℃ (VIF = 17.4) were excluded due to severe multicollinearity (VIF > 10). The refitted final model included postoperative pneumonia, WBC counts, and positive oral secretion culture, all with acceptable collinearity (VIF < 3.2). Logistic regression analysis (Table 3) identified postoperative pneumonia as the sole independent risk factor for EF (*p* = 0.029). Compared with patients without pneumonia, patients with pneumonia had an OR of 5.25 (95% CI, 1.18–23.28; *p* < 0.05). No other variables significantly affected EF (*p* > 0.05).

**Table 3:** Multivariable logistic regression analysis of risk factors for extubation failure.

Variables	Odds ratio	95% confidence interval	*p* value
Postoperative pneumonia	5.25	1.18–23.28	0.029
Postoperative white blood cell count <4 or ≥12 × 10^9^/L	0.39	0.24–2.77	0.751
Positive culture of oral secretions	2.22	0.38–13.18	0.379

### 3.3 Association of EF with short-term outcomes

Patients with extubation failure required prolonged mechanical ventilation (median 5 days [IQR 4–7] vs. 4 days [IQR 4–5]; *p* < 0.001), extended PICU stay (median 9 days [IQR 8–13] vs. 6 days [IQR 5–6]; *p* < 0.001), and greater hospital LOS (median 18 days [IQR 14–22] vs. 15 days [IQR 13–17]; *p* = 0.001) compared with successful extubation (Table 4).

**Table 4:** Comparison of outcomes between extubation success and failure populations.

Variables	Extubation success	Extubation failure	*p* value
	(*n* = 108)	(*n* = 27)	
Days of mechanical ventilation (median, IQR)	4 (4–5)	5 (4–7)	<0.001
PICU length of stay, days (median, IQR)	6 (5–6)	9 (8–13)	<0.001
Hospital length of stay, days (median, IQR)	15 (13–17)	18 (14–22)	0.001

IQR, interquartile range; PICU, pediatric intensive care unit.

## 4 Discussion

In this study, we found that postoperative pneumonia was the most significant risk factor for predicting EF after MDO, while mandibular traction distance before extubation, postextubation laryngeal edema, oral secretion culture positivity, and inflammatory markers (WBC, CRP, temperature) did not accurately predict EF risk. We also found that EF was correlated with an extended period of mechanical ventilation, a prolonged stay in the PICU, and an increased length of hospital stay.

Heterogeneous definitions of EF exist in the current literature (14, 15). Some previous studies have defined EF as the need for noninvasive ventilation or reintubation within 24 h of planned extubation (11, 14). Alternatively, some studies have defined EF as need for reintubation within 48 h of planned extubation (15). Although there are many different definitions, we chose the first definition because all infants with EF needed invasive or non-invasive ventilation and all extubation failures occurred within 24 h in our study.

The post-operative protocol is the same for all patients, including: First, all patients underwent an identical postoperative distraction protocol. Second, all patients followed the same ventilator weaning and extubation strategy. Third, all patients routinely received nebulized epinephrine after extubation to reduce laryngeal edema. Fourth, all patients who experienced respiratory deterioration following postoperative extubation were managed with an identical protocol, including initial nebulization and suctioning; if respiratory distress persisted or worsened, escalation to non-invasive or invasive mechanical ventilation was undertaken. Our study showed postoperative pneumonia was an independent risk factor for EF after MDO in infants with PRS. Zhang et al. also reported that pulmonary infection was an independent predictor of hypoxemia in PRS infants who underwent MDO, but they studied pulmonary infection during ventilator weaning, while the pneumonia discussed in our study occurred during the postoperative phase (9). Postoperative pneumonia is common, whereas pneumonia occurring at the time of ventilator weaning is uncommon. Therefore, our study is more consistent with clinical practice. The following is the possible reason for EF in infants with postoperative pneumonia: Wheezing was frequently auscultated within the first several hours after extubation in infants who failed extubation, suggesting persistent small airway inflammation during this immediate post-extubation period. Pneumonia-associated inflammation of the distal airways (small bronchi and bronchioles) produces edema and luminal narrowing. In this context, even low levels of PEEP (4–5 cmH_2_O) may function as a “pneumatic stent,” maintaining small airway patency and thereby masking underlying respiratory compromise during MV. Although pneumonia has significantly improved in most patients at the time of extubation, residual inflammation in the distal airways may persist. Removal of PEEP at extubation can precipitate a marked increase in distal airway resistance secondary to this residual inflammation, leading to respiratory decompensation. This pathophysiology is consistent with the observation by Ferguson et al., who noted that a majority of extubation failures stemmed from insufficient gas exchange caused by dysfunction in the lower respiratory tract (11). Therefore, prospective studies are needed to evaluate whether screening for postoperative pneumonia and evaluating airway resistance before extubation can reduce the risk of EF.

Insufficient distraction length may prevent relief of tongue-based airway obstruction, ultimately resulting in EF. Previous studies have identified a minimum distraction length of 5 mm for preventing EF after MDO (9, 16). Zhang et al.'s study reported that postoperative mean distraction of 5 mm appeared to be associated with successful extubation after MDO (16). Zhang et al.'s study showed that the length of distraction less than 5 mm at weaning was an independent risk factor for hypoxemia after extubation (9). In our cohort, all infants were extubated with distraction length of ≥5 mm, precluding inadequate distraction as a cause of EF. Paradoxically, patients with EF had longer distraction lengths at extubation. This observation does not reflect a causal relationship, as greater distraction length cannot precipitate respiratory failure (17, 18). Rather, prolonged mechanical ventilation secondary to postoperative pneumonia may facilitate greater distraction lengths following MDO, potentially accounting for the longer distraction lengths observed in EF cases (19).

There have been many controversies over whether laryngeal edema is a main cause of EF (12, 20, 21). Most studies show that laryngeal edema is not a risk factor for EF (22), but a few studies suggest that laryngeal edema is an important cause of EF (23). Consistent with the majority of evidence, our finding indicated that laryngeal edema was not associated with EF following MDO in PRS patients. In clinical practice, post-extubation stridor is frequently observed in pediatric patients with EF, often leading clinicians to erroneously attribute EF to laryngeal edema. However, our results indicated that isolated laryngeal edema did not independently cause EF in the absence of pneumonia.

Multiple studies have shown that EF is associated with serious negative consequences, such as a higher mortality rate, extended duration of PICU and overall hospitalization, and greater healthcare expenditures (24, 25). However, prior studies have not specifically focused on patients with PRS. Our study showed that EF was associated with short-term adverse outcomes in this cohort, including extended mechanical ventilation, critical care stay, and total hospitalization, consistent with previous reports. These adverse outcomes may be attributable to diverse mechanisms, including the direct consequences of reintubation and the clinical deterioration that occurs from the time of extubation until reintubation (26).

This study has some limitations. First, as a single-center retrospective study, there may be an admission bias. Therefore, it is essential to conduct large-scale prospective comparative trials or multicenter study to better assess the risk factors of EF. Second, the multivariable analysis was limited by the relatively small number of extubation failure events (*n* = 27). To optimize model validity, we employed strict univariable screening (*p* < 0.10) and excluded CRP and maximum temperature from the final model due to severe multicollinearity (VIF > 10). The final model included three independent variables with acceptable EPV ratio (9:1) and no multicollinearity (all VIF < 3.2). Nevertheless, our findings should be interpreted as hypothesis-generating and require validation in larger prospective studies. Third, the SBT was conducted by using a ﬂow-inﬂating anesthesia bag. A fresh gas ﬂow was used to prevent rebreathing of exhaled carbon dioxide during spontaneous ventilation, but the oxygen concentration in the fresh air was 100% which is too high to accurately judge whether the lung injury would cause EF (10). Alternative SBT protocols have been shown to improve extubation success rates in pediatric populations. For instance, Faustino et al. validated an extubation readiness test that successfully predicted extubation outcomes in children with acute respiratory failure secondary from lower respiratory tract disease (27). Adoption of similarly validated SBT protocols may enhance extubation success in PRS infants with pulmonary and lower airway diseases.

## 5 Conclusions

In this retrospective study, EF occurred in 20% of infants with PRS following MDO. Postoperative pneumonia was independently associated with EF, with a fivefold increase in risk on the first extubation attempt. Prospective studies are needed to evaluate whether screening for postoperative pneumonia and evaluating airway resistance before extubation can reduce the risk of EF. Mandibular distraction length, laryngeal edema and some inflammatory markers were not associated with EF. EF was associated with extended duration of mechanical ventilation, longer PICU and hospital LOS in these infants.

## Data Availability

The raw data supporting the conclusions of this article will be made available by the authors, without undue reservation.
